# Biosafety in Dental Health Care During the COVID-19 Pandemic: A Longitudinal Study

**DOI:** 10.3389/froh.2022.871107

**Published:** 2022-05-10

**Authors:** Lucyene Miguita, Roberta Rayra Martins-Chaves, Victor Emmanuel Viana Geddes, Suellen da Rocha Mendes, Sara Ferreira dos Santos Costa, Paula Luize Camargos Fonseca, Diego Menezes, Rafael Marques de Souza, Daniel Costa Queiroz, Hugo José Alves, Raphaela Alvarenga Braga de Freitas, Aline Fernanda Cruz, Rennan Garcias Moreira, Filipe Romero Rebello Moreira, Larissa Marques Bemquerer, Diego Rodrigues de Aguilar, Maria Elisa de Souza e Silva, Aline Araújo Sampaio, Francisca Daniele Moreira Jardilino, Leandro Napier de Souza, Tarcilia Aparecida da Silva, Carolina Cavaliéri Gomes, Mauro Henrique Nogueira Guimarães de Abreu, Renato Santana de Aguiar, Renan Pedra de Souza, Ricardo Santiago Gomez

**Affiliations:** ^1^Department of Pathology, Biological Sciences Institute, Universidade Federal de Minas Gerais (UFMG), Belo Horizonte, Brazil; ^2^Department of Oral Surgery and Pathology, School of Dentistry, Universidade Federal de Minas Gerais (UFMG), Belo Horizonte, Brazil; ^3^Department of Genetics, Ecology and Evolution, Biological Sciences Institute, Universidade Federal de Minas Gerais (UFMG), Belo Horizonte, Brazil; ^4^Department of Community and Preventive Dentistry, School of Dentistry, Universidade Federal de Minas Gerais (UFMG), Belo Horizonte, Brazil; ^5^Multiusers Laboratories Center, Biological Sciences Institute, Universidade Federal de Minas Gerais (UFMG), Belo Horizonte, Brazil; ^6^Departamento de Genética, Instituto de Biologia, Universidade Federal do Rio de Janeiro (UFRJ), Belo Horizonte, Brazil; ^7^Department of Operative Dentistry, School of Dentistry, Universidade Federal de Minas Gerais (UFMG), Belo Horizonte, Brazil

**Keywords:** COVID-19, SARS-CoV-2, RT-PCR, antibodies, variant, dental public health

## Abstract

**Background:**

The coronavirus disease 2019 (COVID-19) pandemic had quite an impact on dental health care. Concerns about the risk of SARS-CoV-2 transmission through contaminant fluids and droplet formation during several dental procedures highly impacted dental health care, drastically reducing the number of dental practices worldwide. To monitor SARS-CoV-2 contamination in dental clinics, a longitudinal study was carried out during the return of dental practice at university.

**Methods:**

Dental health care professionals [(DHCPs); teachers, undergraduate dental students, and dental assistants] and patients were screened for SARS-CoV-2 RNA in a dental school clinic environment from 11^th^ January to 12^th^ March 2021 (9 weeks). Serological testing was performed on DHCPs in two-time points. Additionally, samples with low *Ct* values were sequenced to identify the circulating SARS-CoV-2 variant and possible transmission clusters.

**Results:**

We found a low number of dental staff (5.8%), patients (0.9%), and environment sites (0.8%) positive for SARS-CoV-2. Most positive cases had asymptomatic to mild symptoms, and two asymptomatic DHCPs presented prolonged infection. In the first week after previous exposure to COVID-19, 16.2% of DHCPs had IgM or IgG antibodies against SARS-CoV-2, and 1/3 of them had undetected antibodies in the last weeks. The variant zeta (P.2) could be detected. No cross-infection was observed between participants.

**Conclusion:**

Our study suggests that dental practice can be safely executed when adequate control measures and biosafety protocols are applied. DHCP and patient testing, patient telemonitoring, proper use of personal protection equipment, and sanitization of surfaces are essential to avoid SARS-CoV-2 cross-infection in dental practice.

## Introduction

The coronavirus disease 2019 (COVID-19) outbreak resulted from severe acute respiratory syndrome coronavirus 2 (SARS-CoV-2), which triggers a systemic disease with heterogeneous clinical manifestations, from asymptomatic to multiorgan failure [1], causing substantial health impacts in several countries, negatively affecting dental care.

As dentists work in close contact with patients, initial studies have shown potential increasing risks related to dental practice, both for dental staff and patients [2, 3]. The transmission of SARS-CoV-2 is mainly due to inhalation or direct contact with contaminated fluids, including saliva droplets. This pathogen can also survive on solid surfaces exposed to contaminated fluids [4–7].

To reduce the risk of contamination in dental practice, in April 2020, the American Dental Association (ADA) and the Center for Disease Control and Prevention (CDC) recommended that dental healthcare professionals (DHCPs) conduct only urgent and emergency procedures, avoiding any routine dental care that could generate aerosols [8].

Since then, many private and public dental clinics have stopped or reduced the number of appointments due to scarce personal protective equipment (PPE) availability and adapted to the facilities and protocols [9–11], increasing the number of dental emergencies [12] and affecting dental education [13, 14]. The changes suggested by health and professional agencies are significant. However, it is necessary to assess their effectiveness as preventive and protective measures against COVID-19 in the return of clinical dental practice.

In this present study, to monitor contamination of SARSCoV-2 in dental clinics during the return of students to university, a longitudinal study was carried out evaluating the efficacy of constant testing in environment, teachers, dental students, dental assistants, and biosafety protocols implementation to prevent SARS-CoV-2 transmission during the return of dental practice at university.

## Materials and Methods

### Ethical Approval and Consent of Participants

The present study was approved by the Ethics Committee of Universidade Federal de Minas Gerais (Protocol CAAE n°31041720.3.0000.5149). All participants enrolled in this study were volunteers, and their samples and clinical data were collected only *via* signed consent forms.

### Study Design

**Figure 1:**
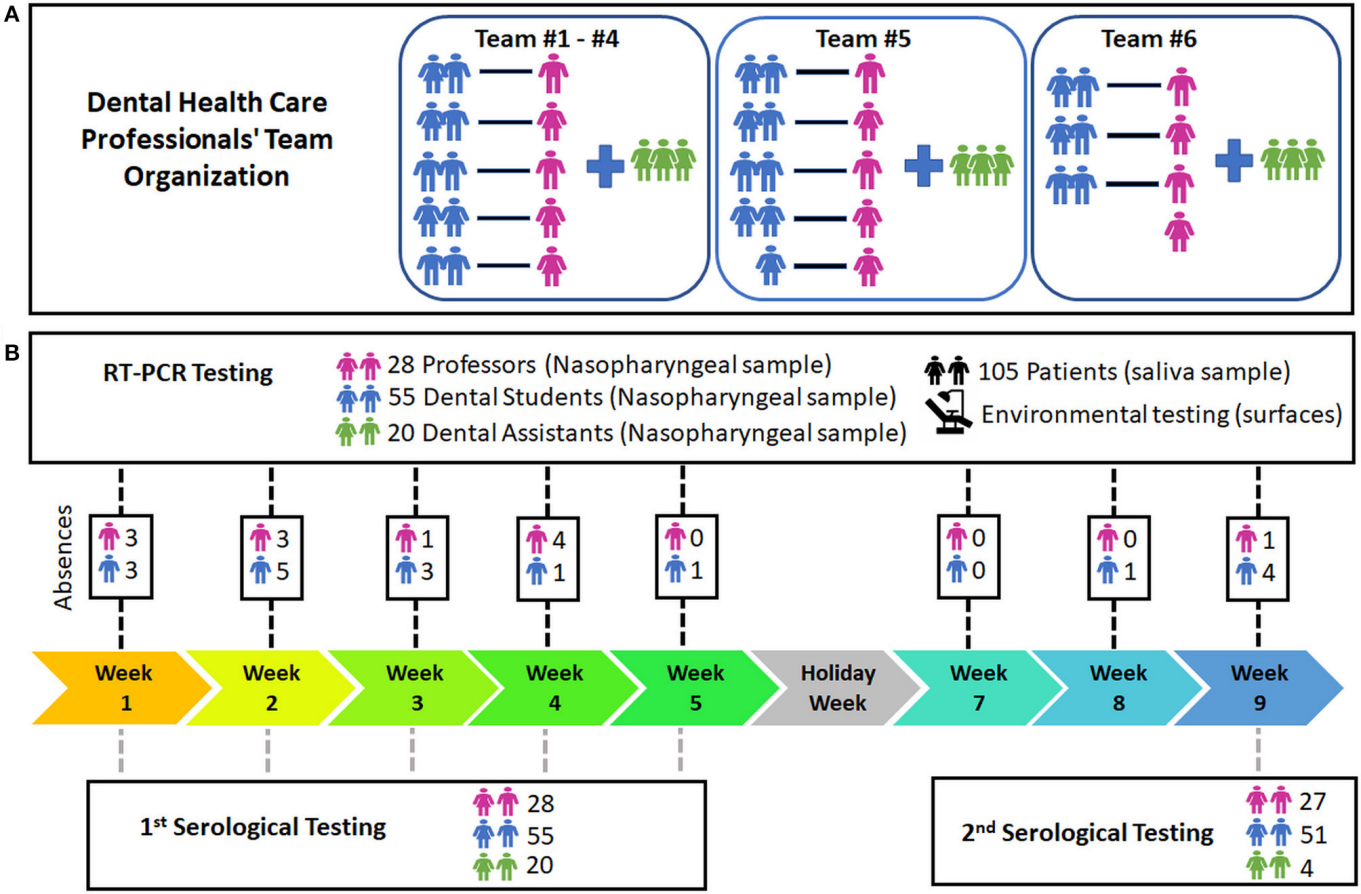
Study workflow and dental healthcare professionals' team. **(A)** Teams of dental healthcare professionals. **(B)** Testing workflow of the study. The presence and absence of each dental healthcare professional per week are demonstrated on Supplementary Figure 5.

A longitudinal study with convenience sampling was performed at the Clinic of Emergence at the School of Dentistry of UFMG, from 11th January to 12th March 2021 (9 weeks) (Figure 1). All DHCPs (*n* = 103) were trained before following new dental care protocols by ADA/CDC/ANVISA for COVID-19 [8, 15] and presented a knowledge test with a minimum passing score of 80% or more. All patients (*n* = 105) were previously telemonitored and only participants presenting body temperature measured below 37°C have access to the dental clinic, according to ADA/CDC/ANVISA recommendations [8, 15], and filled a metadata form (Supplementary Figure 1).

In the first and last week of the study, whole blood samples were collected from the DHCPs (teachers, dental students, and dental assistants) to detect anti-SARS-CoV-2 IgM and IgG antibodies using serological tests (732-10, *Labtest Diagnóstica*). Virus RNA was weekly investigated by real-time PCR (RT-PCR) in nasopharyngeal samples from DHCPs and saliva samples from patients attending the clinic.

The environmental sampling was tested by RT-PCR and performed on day one, before the first day of activities, and once a week after dental procedures.

The personnel who collected the samples were also monitored weekly using nasopharyngeal swabs and RT-PCR. Only those who tested negative participated in sample collection (data not shown).

### Sample Collection From Dental Health Care Professionals, Patients, and Environment

Nasopharyngeal swabs were collected by trained investigators and maintained in a 0.8-ml viral transport medium (VTM). Up to 3.2-ml volume of non-stimulated saliva samples were collected in 50 ml sterile tubes before analysis. Up to 10 nasopharyngeal samples and 3–5 saliva samples were pooled and analyzed by RT-PCR [16].

The environmental sampling was collected in 6 main areas (Supplementary Figures 2–4), totaling 100 sites from frequently touched surfaces, surfaces near 1–2 m distance from dental chair, and air. A sterile swab embedded in VTM was used to collect samples from a minimum of 25 cm^2^ area of each surface. Sampling from the internal part of the dental suction system was performed with swab introduction (approximately 20 cm in length). A tube containing the VTM was kept open during the whole procedure of environmental sampling.

### RNA Extraction and RT-PCR

Molecular diagnosis was performed in accordance with the CDC 2019-Novel Coronavirus (2019-nCoV) Real-Time RT-PCR Diagnostic Panel. Viral RNA extraction was performed using the Quick-RNA™ Viral Kit (R1035, ZYMO Research) and amplified using the Multiplex Luna® Universal Probe One-Step RT-PCR Kit (New England Biolabs, Bioscience) and 2019-nCoV RUO kit (10006713, IDT) for *N1, N2*, and *RNase P* gene regions. Reactions were performed using an Applied ABI 7500 (Applied Biosystems). Positive and negative controls were used in each run to validate the method, including standard curves. When pooled sample amplified SARS CoV-2 *N1* and/or *N2* genes with cycling threshold (Ct) values minor 40, the pooled samples were individually diagnosed.

### Whole Virus Genome Sequencing

All positive samples (*N1* or *N2* targets, *Ct* < 30) were sequenced using the QIAseq FX DNA Library Prep kit (QIAGEN, Germany) and the Illumina MiSeq (Illumina, USA). Negative control was included, and a custom pipeline for data quality control and consensus genome reconstruction was used [17]. All mutations detected in the novel consensus genome were manually verified. The viral genomes were classified into Pango lineages (pangolin tool v2.4.2). To corroborate the classification, a dataset (*n* = 103) containing only lineages identified in Belo Horizonte during January and February 2020 was created using public genomes (GISAID EpiCoV database). The dataset was aligned (Minimap2 [18] and a maximum likelihood phylogeny was inferred (Q-tree v2.0.3 [19] - GTR+F+I+G4 model [20, 21].

### Data Analysis

Categorical data were presented using absolute and relative frequencies. Numerical data were presented using mean and standard deviation. All estimates were calculated using Microsoft Excel.

## Results

### SARS-CoV-2 Infection Prevalence in Dental Health Care Professionals Using RT-PCR

Before the study period, 48.5% (50/103) of the participants reported being tested by different types of COVID-19 test, and 12.6% (13/103) of them tested positive. Among these 13 DHCP reporting previous positive tests, there were nine students, three dental assistants, and one teacher (Tables 1, 2).

**Table 1:** Demographic and clinical data of participants.

	**Teachers**	**Dental students**	**Dental assistant**	**Patients**
**Total (n)**	28	55	20	105
**Age**				
Mean (±PD)	43.4 (±7.2)	25.6 (±2.8)	50.2 (±10.5)	44.8(±17.1)
**Sex**				
Female (%)	14 (50.0%)	43 (78.2%)	20 (71.4%)	75 (71.4%)
Male (%)	14 (50.0%)	12 (21.8%)	8 (28.6%)	30 (28.6%)
**Comorbidities/conditions**				
Pregnant or breastfeeding	–	1	1	2
Hypertension	1	–	4	29
Diabetes	–	–	1	9
Immunodepression	–	–	–	1
Lung disease	1	4	1	6
Heart disease	–	–	–	4
Kidney disease	–	—	-	1
Liver disease	-	-	-	-
Other	–	1	–	16
**Symptoms in recent days**				
Fever	–	1	1	–
Shortness of breath	1	3	1	4
Chills	1	2	2	1
Diarrhea	2	9	1	–
Loss of taste	–	4	4	4
Tiredness or fatigue	2	8	2	3
Cough	5	4	3	5
Headache	6	17	12	9
Sore throat	1	4	5	4
Decreased smell	–	2	4	2
Muscle or body aches	1	8	4	4
Other	–	–	–	2
**Time of onset of symptoms**				
Less than 7 days	–	8	3	6
Between 7 and 14 days	–	3	2	4
Between 15 and 21 days	1	1	2	–
More than 21 days	8	12	6	12
**Previous COVID-19 testing**				
Yes	17	26	6	17
No	11	27	21	88
**Exam type**				
Immunochromatography serological test	2	4	–	–
Chemiluminescence serological test	–	2	–	1
Fluorescence serological test	1	–	–	1
ELISA serological test	3	1	3	1
Molecular test (RT-PCR)	15	22	4	9
Did not know how to inform			1	4
**Test result for COVID-19**				
Positive	1	9	3	5
Inconclusive	–	–	–	–
Negative	16	18	3	11
Another vírus	–	–	–	–

*n, number. PD, Pattern Deviation. %, percentage*.

**Table 2:** History of travel and previous contact with a COVID-19 positive person.

	**Teachers**	**Dental students**	**Dental assistant**	**Patients**
**Total (** ***n*** **)**	28	55	20	105
**Contact with confirmed or suspected COVID-19 case**				
Yes	12	30	13	11
No	16	25	15	94
**Contact period**				
Less than 7 days	–	7	5	1
Between 7 and 14 days	2	2	2	2
Between 15 and 21 days	1	2	–	–
More than 21 days	9	19	5	7
**Contact with symptomatic case?**				
Yes	5	21	9	7
No	4	6	1	2
Did not know how to inform				
**Contact with confirmed case?**				
Yes	10	22	9	7
No	2	3	2	1
Did not know how to inform	–	–	–	–
**Exam type (Contact)**				
Immunochromatography serological test	2	1	1	–
Chemiluminescence serological test	1	1	–	1
Fluorescence serological test	–	–	1	–
ELISA serological test	–	–	3	–
Molecular test (RT-PCR)	6	23	3	4
Did not know how to inform	1	5	5	5
**Have you traveled anywhere in the past few days?**				
Yes, to other City in MG State	5	12	3	7
Yes, to other Brazilian State	8	7	2	3
Yes, to other Country	1	2	–	–
No	14	34	23	95
**If you answered yes to the previous question, what is the return time for the trip?**				
Less than 7 days	6	8	1	1
Between 7 and 14 days	5	10	4	1
Between 15 and 21 days	1	1	-	2
More than 21 days	1	-	-	5
**Have you been vaccinated recently?**				
Yes	3	6	4	7
No	25	48	24	98
**Recent vaccination type**				
Flu Vaccine	1	1	2	6
Pneumonia Vaccine (Pneumococcal vaccine polyvalent)	–	–	–	–
Other	2	5	2	1
**Period of vaccination**				
Less than 7 days	–	–	–	–
Between 7 and 14 days	–	1	–	1
Between 15 and 21 days	–	1	1	–
More than 21 days	3	4	3	6

*n, number*.

During the study, 5.8% (6/103) of the DHCP tested positive for SARS-CoV-2 (one teacher, three students, and two dental assistants).

**Figure 2:**
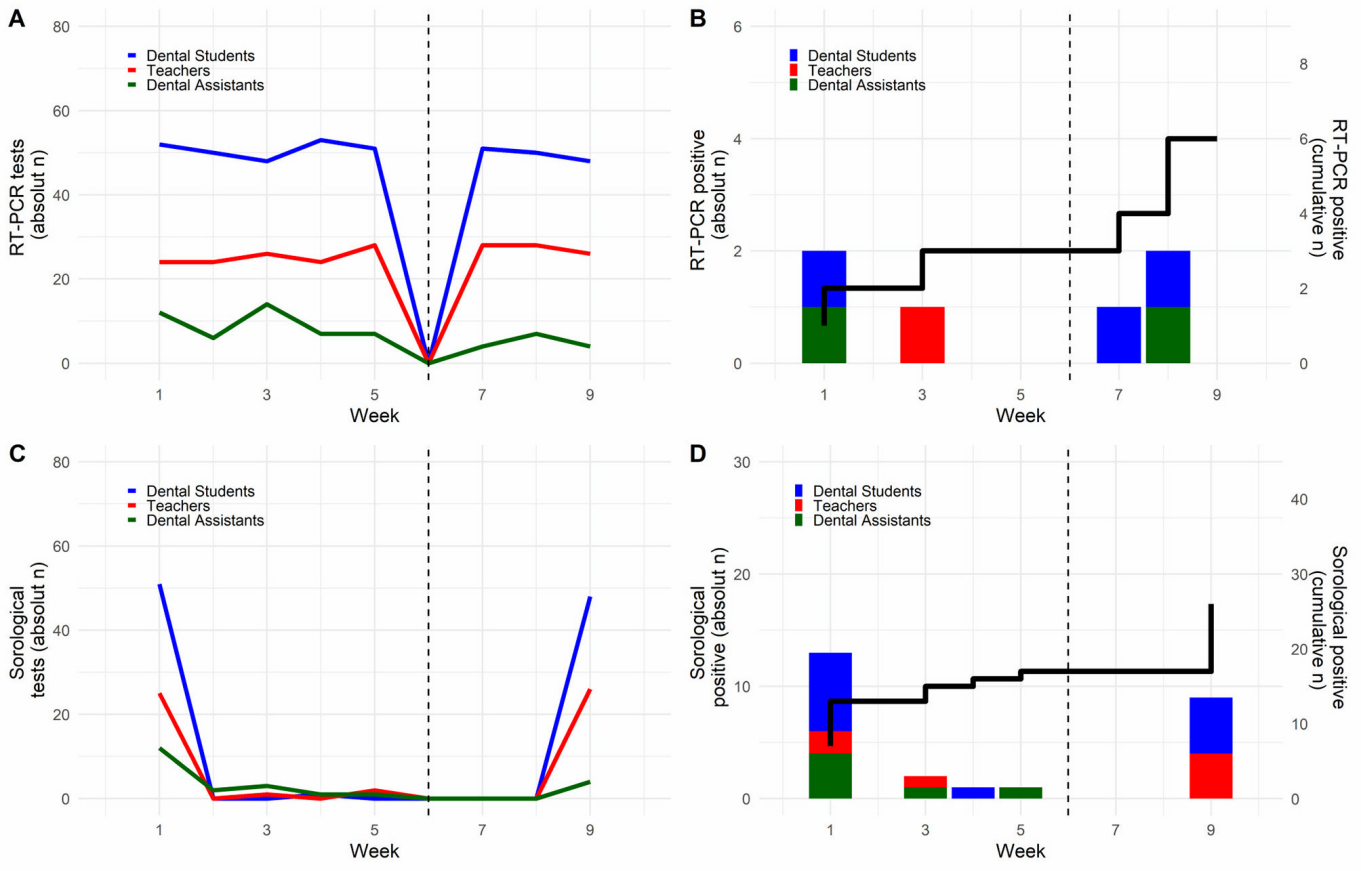
RT-PCR and serological tests in dental healthcare professionals. **(A,B)** Shows the number of RT-PCR tests and positive results, respectively. The black line shows cumulative cases **(C,D)** present the number of serological tests and the positive results of each week of the study. The black line shows cumulative cases.

Timelines with the number of RT-PCR tests and positive results per DHCP are illustrated in Figures 2A,B, respectively. According to the presence and week, all DHCPs' results are shown in Supplementary Figure 5.

Indeterminate results (i.e., *Ct* <40 for *N1* or *N2* genes) are indicative of lower viral load during the beginning or end of viral peak and represented a total of 16.5% in our study (Supplementary Figure 5). These individuals were retested the following week and found to be negative. Only one student with an indeterminate test was symptomatic and tested negative 14 days later, indicating that the infection had been resolved.

### Asymptomatic Prolonged Infection Cases

Asymptomatic prolonged infections were identified in two individuals. A teacher (L#10) tested positive three times (on weeks 3, 5, and 9), after the first RT-PCR positive result in the study, with an interval of 15 and 43 days. A student (S#52) tested positive two times (weeks 1 and 7), with a 49-day interval between each positive result. Both were female, IgG positive in the second serological tests, and reported no symptomatology when RT-PCR results were positive.

### Prevalence of Antibodies Against SARS-CoV-2 in Dental Health Care Professionals

On the first serological testing, 99 DHCPs were analyzed and 16.2% (16/99) presented positive results. Of those, 3.0% (3/99) were IgM+ only, 8.1% (8/99) were IgG+ only, and 5.1% (5/99) were IgM+/IgG+. Only one participant was immunized for COVID-19 during the study.

At the final antibody testing, a reduced number of dental assistants and students (totaling 76/99; 76.8%) were present in the clinic due to work schedule and graduation course completion, respectively and 15.8% (12/76) of the participants tested positive (4 IgM+, 4 IgG+, and four positive for both IgM and IgG).

Considering the two time-points of serological testing, 7.9% (6/76) remained positive results in both tests. Out of this, four of them maintained the same serology (2 IgM+, 1 IgG+, and 1 IgM+/IgG+). The other two presented IgM antibodies in the beginning and IgM+/IgG+ in the second test; one reported to have contact with a confirmed COVID-19 case and the other received the COVID-19 vaccine before the serological test. All six participants were negative for the RT-PCR tests during the whole study, suggesting previous exposure to SARS-CoV-2 and COVID-19 vaccine.

In total, 5.3% (4/76) DHCPs had antibodies detected at the beginning of the study but no longer showed positivity after 2 months, presenting the second serological result negative for both antibodies. These four individuals tested negative in all RT-PCR tests.

During the study, seroconversion was observed in the two prolonged infection cases. They were negative for antibodies at the beginning of the study and presented IgG antibodies at the end, confirming the exposure to COVID-19 during the study.

Figures 2C,D show the absolute number of serological tests performed in each group of dental staff and positive results. Supplementary Figure 6 shows the number of positive IgG and IgM antibody positive cases and cumulative cases are represented by the black line.

### Co-worker Infection Assessment

**Figure 3:**
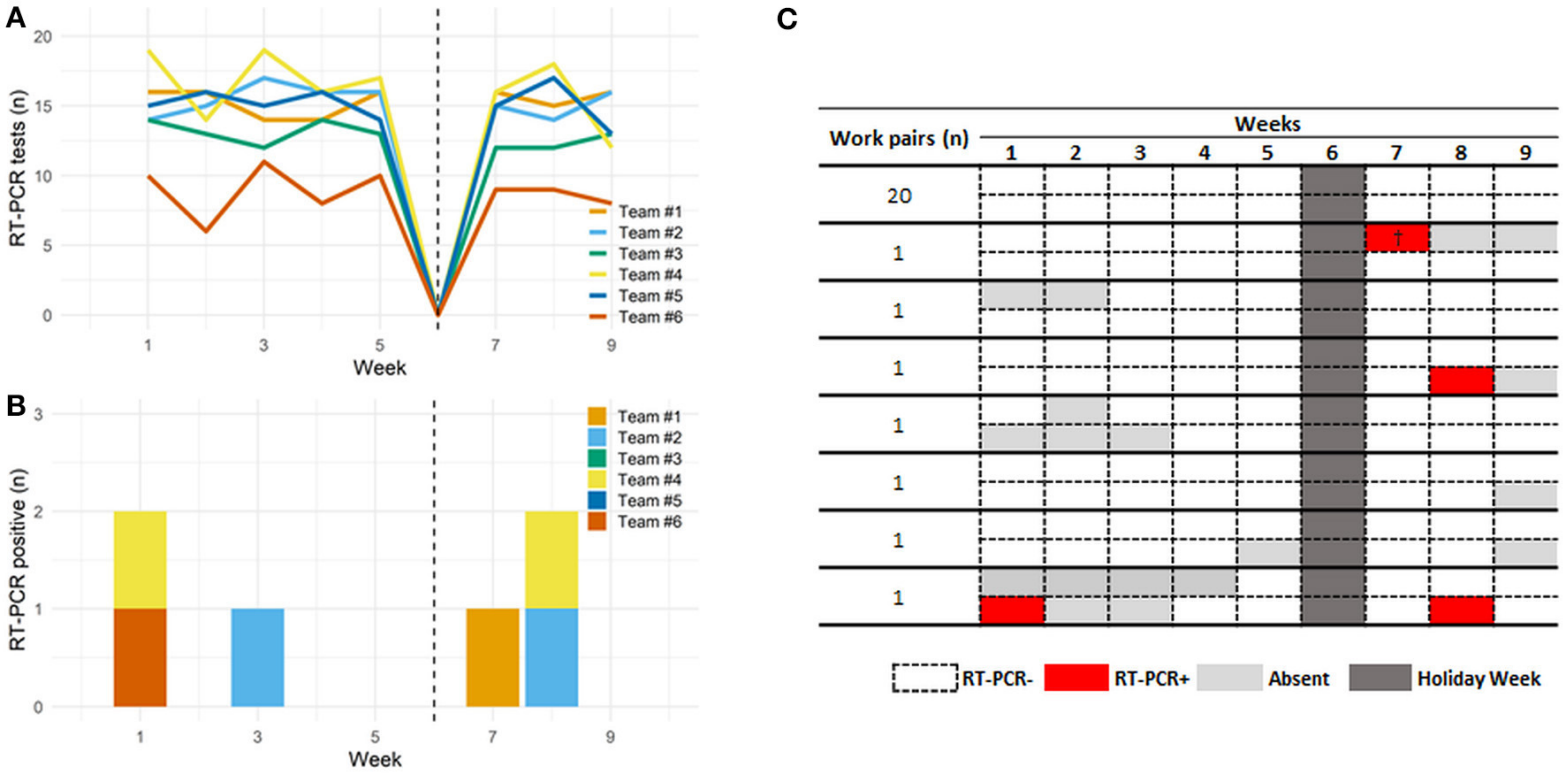
RT-PCR tests and results for SARS-CoV-2 in dental healthcare professionals. **(A)** Shows the number of RT-PCR tests during study timeline per team composed by teachers, dental students and dental assistants. The number varied between teams due to the absence of some participants, either because of COVID-19 diagnostic or personal reasons. **(B)** Shows the number of RT-PCR positive cases by team during the 9 weeks. **(C)** Shows the presence and results found in work pairs of dental students. (†), shows the sequenced samples of the SARS-CoV-2 zeta variant.

In order to evaluate whether the measures to control COVID-19 transmission implemented in this study were efficient, we analyzed possible co-worker infections. We evaluated teams composed of teachers that individually monitored work pairs of dental students (Figure 1A). Each team was present in one fixed period of a weekday in the dental clinic and 12.9±3.1 RT-PCR tests were performed weekly per team (Figure 3A). The number of positive cases per week is presented in Figure 3B.

The students worked with the same partner throughout the study, and 27 students' work pairs were evaluated. No cross-infection was detected in either partner, neither simultaneously nor one following another, in the subsequent weeks (Figure 3C and Supplementary Figure 5).

### Prevalence of SARS-CoV-2 in Asymptomatic Patients

A total of 105 patients participated in the study (Tables 1, 2). Only one patient (1/105; 0.9%) was positive for SARS-CoV-2 on the 8th week and was an asymptomatic boy.

Most patients were only tested once because they did not have to return for dental procedure follow-up. Therefore, 18.1% (19/105) of the patients could be retested (for 2–3 weeks), and all retested negative, reinforcing that all the control procedures prevented the infection of patients during the dental practice.

### Environmental Testing

**Figure 4:**
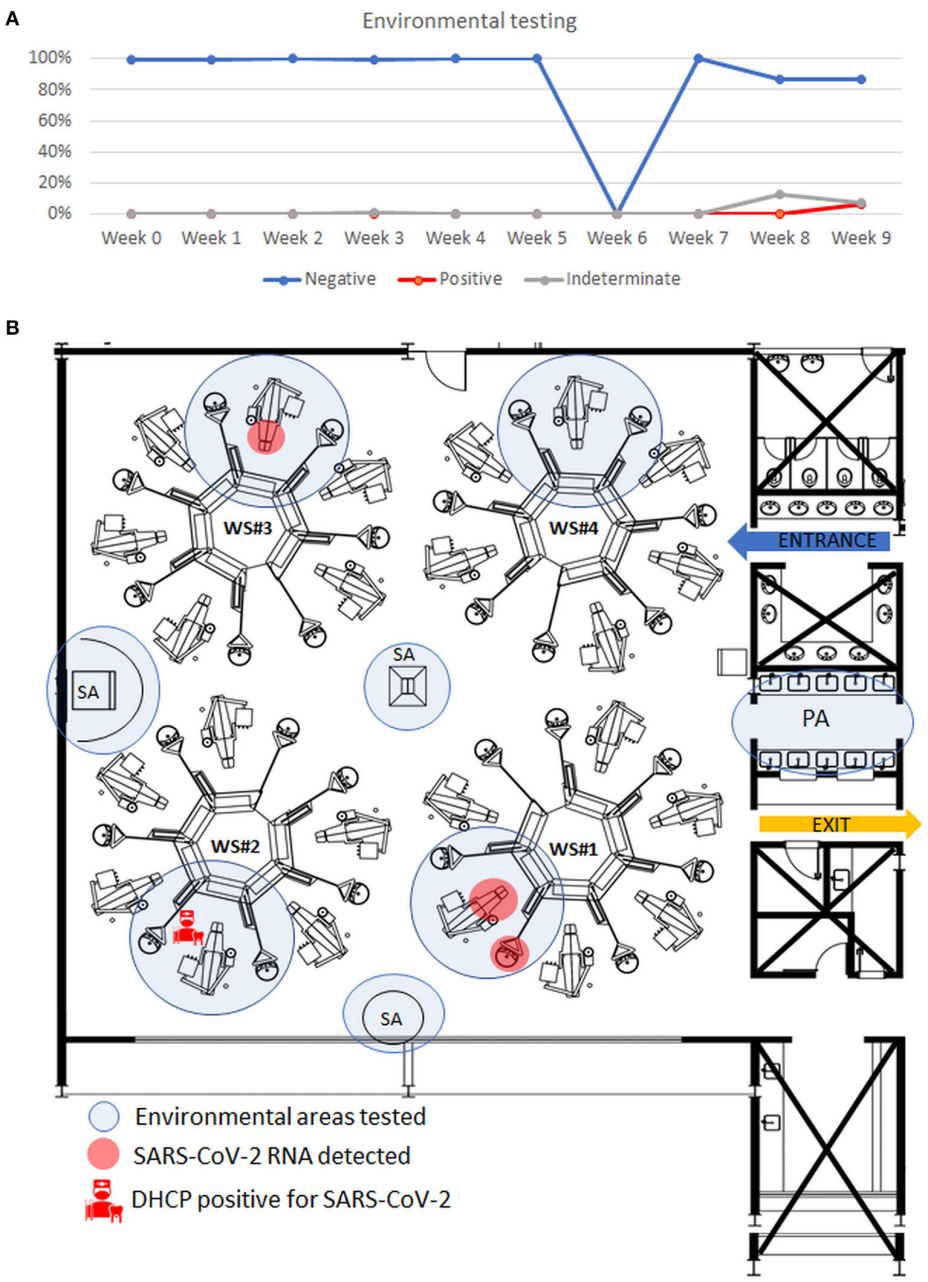
Results of environmental samples collected from the dental clinic and tested by RT-qPCR. **(A)** Graphic representation of the results of environmental samples per week. Values in percentage. **(B)** Representative figure, on week 9, showing the spatial distribution of areas positive for SARS-CoV-2 RNA, detached in red, and position of the detected positive dental health professional in the workstation#2 in the clinic. Workstation #1 presented detected (the sink bench, detergent dispenser, dental chair upholstery, light handle, and light arm) and indeterminate results (non-hazardous waste disposal, air sample, and saliva ejector hose–external part). The workstation #3 presented one sample positive in the light arm and presented indeterminate results in the other three spots. WS, workstation. DHCP, dental health care professional. PA, Purge area. SA, supporting area.

A total of 898 samples were collected (Figure 4A). Positive samples (0.7%, 6/898) were found only in the 9th week (Figure 4B) and indeterminate samples (2%, 21/898) were found on the 8th week and 9th weeks (Supplementary Figure 7).

Comparing the 2 weeks, the positive and indeterminate surfaces were different each week. The supporting and purge areas were negative in all the weeks tested (Supplementary Figure 7).

### SARS-CoV-2 Variants Identified

To evaluate whether the control protocols prevent cross-infection among the participants, we sequenced the whole genome of SARS-CoV-2 from two positive samples presenting *Ct* values compatible with whole genome sequencing. Both participants (student and dental assistant) never worked at the same work team over the entire study and became positive in different stages of the study: the dental assistant (LBI_279) became positive at the first week and the student (LBI_283) at the 7th week, suggesting no possible cross-infection at the clinics. The phylogenetic reconstruction ruled out the possibility of virus spillover during the clinics and cross-infection between the two participants (Supplementary Figure 8). Both viral genomes are classified as zeta variant (previous P.2), the most prevalent SARS-CoV-2 lineage present in the city during the study. However, each sample was grouped in different branches compared to other virus references sequences from the Belo Horizonte city at the same time of the study reinforcing that both cases came from two independent events of infection unrelated to the dental clinical practice.

## Discussion

The study was conducted at the beginning of a big wave of COVID-19 in Belo Horizonte City and the vaccination distribution was limited to a few health care workers at hospitals. In this period, only 1.3% of the Brazilian population was vaccinated. This scenario made it possible to realize the study in a convenience sample of DHCPs and patients of a public dental health care University from Brazil. The presence of the COVID-19 virus has been demonstrated in oral tissues and saliva [4, 6]. This triggered concerns about biosafety in dental practice, how to detect and manage patients with COVID-19 when they need dental and oral lesion assistance, and controlling and minimizing the virus cross-infection.

To analyze the biosafety of dental treatment during the COVID-19 pandemic period, a longitudinal screening for SARS-CoV-2 was conducted on DHCPs, patients, and the environment by RT-PCR test during 9 weeks of follow-up. Additionally, two time-points of serological testing and identification of SARS-CoV-2 variants were also performed. To the best of our knowledge, this is the first study in dental health care that experimentally addresses all these points.

During the study, detection of antibody levels for SARS-CoV-2 was present in 16.2% DHCPs; this number was similar to the seroconversion rate (16.3%) found in dental healthcare workers reported by Shields et al. in 2021 [22]. Half of the DHCPs sustained the positive serology results during the 2 months of our research. IgM and IgG levels are known to decrease over time significantly. While IgM decreased by 53%, IgG decreased by 32%, and the number of the receptor-binding domain (RBD)-specific memory B cells could be detected 6.2 months after infection [23].

The RT-PCR and serological tests were suitable methods to detect and alert patients and DHCPs about the need to implement preventive measures during the daily life of participants. We observed a low number of DHCPs who tested positive for SARS-CoV-2 by RT-PCR during the study (6%). Notably, most of the positive and indeterminate results were observed in the week following the *carnival* holiday. It is known that secondary infection by household contacts occurs around 16.3–52.4% [24, 25] when quarantine measures are not respected between individuals [24] and immediately after symptom onset in the first case [25]. In our study, some of these DHCPs reported to have traveled to their family's home cities, and others confirmed contact with someone positive, which is highly suggestive of COVID-19 contamination outside the dental clinic. This fact was also established by the phylogenetic inference of whole genome sequencing from SARS-CoV-2 positive samples. Despite the crescent COVID-19 vaccination and its efficacy in reducing disease severity [26, 27], it is important to monitor asymptomatic individuals and encourage the continued use of preventive measures not only in the dental clinic but also in the social environment to avoid the spread of the disease until total population immunization. In addition, until the end of the study, only 1.3% of the Brazilian population was immunized, demonstrating the efficacy of control measures applied.

The continuous testing allowed the detection of 2 cases of prolonged infection with positive RT-PCR results for 43 and 49 days. Although rare cases of prolonged infection or reinfection, it seems to be related to SARS-CoV-2 intra-host evolution and viral replication that can generate quasi-species diversity [28]. This prolonged infection varies according to host capacity to control infection and may present low transmissibility after the first week of the disease, which is the time when the number of viable virus titles in the upper respiratory tract is at its peak [29, 30].

We found no cross-infection between co-workers or patients, nor a positive environmental area for SARS-CoV-2 RNA where the DHCPs tested positive. This is probably due to the adequate use of PPE to reduce the risk of COVID-19 transmission significantly [31]. It is important to remember that in the present study, all DHCPs were trained for biosafety protocols before the study following the CDC/ADA/ANVISA recommendations [8, 15]. It states the proper use of complete PPE included wearing a disposable isolation gown, N95 respirator, face shield, goggles, disposable cap, gloves, safety glasses, and shoes. All metal, plastic, and marble surfaces were sanitized with 70% ethanol and dental chair upholstery with quaternary ammonium detergent before and after patient assistance; the same workstation was used with an interval of 24 h between each patient. One particular detail of the present dental clinic was the natural ventilation of the environment and the significant distance between workstations (approximately 10 m from each other), which could reduce cross-infection between participants.

Environmental contamination was mainly present during the last 2 weeks of the study. Such positivity was probably due to the secretion of patients who tested positive for COVID-19 during dental treatment. The workstation where the positive patient was assisted presented indeterminate results in some surfaces and air. In the following week, there were positive areas, but not all patients could be tested, and the unique DHCP who tested positive for SARS-CoV-2 in this week worked in a workstation area that tested negative, reinforcing the environmental contamination most probably resulting from the patients' fluids. In the last 2 weeks of the study, the Belo Horizonte City population presented an increasing number of positive COVID-19 individuals, and sanitary measures were more restrictive at this moment due to the gravity of the COVID-19 pandemic. Viable SARS-CoV-2 can be detected on surfaces after hours and not viable viral RNA after days according to the material and in laboratory conditions [5]. In addition, environmental interference, such as temperature, humidity, and heat makes its transmission ability by objects lower than expected in the public environment [7, 21]. Airborne transmission seems to be the dominant route of SARS-CoV-2 transmission [32]. New evidence suggests that smaller droplets present reduced airborne transmission because they carry fewer viruses and evaporate faster than large droplets, causing reduced virus viability in the environment [33].

Since environmental and saliva samples present lower viral loads than nasopharyngeal samples, up to 5 environments and saliva samples were pooled, while up to 10 nasopharyngeal samples were pooled. This technique reduced the cost of testing a large number of samples and efficiently detected positive samples, as described previously [16, 34, 35].

The saliva of all patients tested in the study was collected. The saliva can present viable virion isolation for SARS-CoV-2 [36] because salivary glands seem to be a reservoir of the virus [6]. Saliva is an easy and accessible sample source during dental care and its collection is less uncomfortable than collecting nasopharyngeal samples. Its PCR results present sensitivity (83.2%) and specificity (99.2%) that are very similar to nasopharyngeal samples (84.8% sensitivity and 98.9% specificity) [35]. This sampling technique allowed easy collection of samples from patients, including special care ones and children. In the present study, it was possible to detect an asymptomatic boy of 6-years-old, similar to the previous study that demonstrated a SARS-CoV-2 positive rate of 2.3% in pediatric dental patients, with 50.9% of them being male at a mean age of 6 years and presenting no symptomatology for the disease, suggesting the practice of PCR testing in dental clinic as an adjuvant for screening questionaries [37].

Our study had a limitation in that not all patients visiting the clinics could be tested, and dental assistants were tested only when they were scheduled to be at the University. Most students and teachers were scheduled to be at the clinics every week and had <10% of missing data points. Thus, 76 participants (73.8%) were tested in all weeks of the study. Interestingly, our findings demonstrated that these individuals were not infected after receiving or delivering dental procedures.

Using viral genomics and phylogeny inferences, we showed that positive participants that became positives during the study were infected with different viruses more related to viral genomes from the city of Belo Horizonte than each other. To improve our analysis, we enriched our data set of references sequences with the zeta variant that was the most predominant in the area during the study. The genetic analysis reinforces the efficiency of PPE, constant testing, and environment clean-up to prevent virus spillover events in the dental clinic practice.

In conclusion, this study demonstrated that dental health care assistance possesses a low risk of cross-infection between the DHCPs and patients when biosafety and PPE protocols are adequately followed. Furthermore, our findings show that the infected people present in the clinic were contaminated when socializing with someone contaminated (family/friend) outside the clinic, reinforcing the need to instruct people about social distancing and the importance of using face masks to control the spread of the virus.

## Data Availability Statement

All data that underlie the findings reported on this study (participant's data, after de-identification, tables, figures, appendices, study protocol, cycling threshold results, and informed consent forms), will be available for 5 years, under request by contacting the corresponding authors to researchers or investigators with the sound proposal. Public genomes of LBI_279 and LBI_283 are available on the GISAID EpiCoV database (https://www.gisaid.org/) under the following codes EPI_ISL_1495039 and EPI_ISL_1495042, respectively. The proposal should be directed to the corresponding authors' e-mail.

## Ethics Statement

The studies involving human participants were reviewed and approved by the Ethics Committee of Universidade Federal de Minas Gerais (Protocol CAAE n°31041720.3.0000.5149). All participants enrolled in this study were volunteers, and their samples and clinical data were collected only via signed consent forms. Written informed consent was obtained from the individual(s), and minor(s)' legal guardian/next of kin, for the publication of any potentially identifiable images or data included in this article.

## Author Contributions

The project administration, resources, funding acquisition, and supervision were performed by RG, RPS, RA, and MA. Substantial contributions to the study conceptualization were performed by RG, RPS, RA, MA, CG, and RM-C. Data analysis and curation were executed by LM, SR, SS, and DM. The formal analysis was performed by LM, RPS, RA, and RG. Methodology was contributed by RG, RPS, RA, MA, AS, FJ, LS, TS, and LM. Software analysis was performed by FM and PF. Data validation was performed by FM, RPS, RA, and RG. Visualization was executed by LM, RPS, RM-C, VG, PF, and MS. Original draft and editing of writing were performed by LM, RM-C, CG, VG, RG, RPS, and RA. Data acquisition was contributed by LM, RM-C, VG, SR, SS, DM, RMS, DQ, HA, RF, AC, RM, LB, DA, AS, FJ, LS, TS, and RSG. All authors have read and agreed to the published version of the manuscript.

## Funding

This study was financially supported by UFMG/RTR/PRPq and FAPEMIG (RG, grant n° 00712-20, RPS, grant n° APQ-00475-20), CNPq (RA, grants 312688/2017-2 and 439119/2018-9; RPS, grant n° 310627/2018-4), the Coordination for the Improvement of Higher Education Personnel (MEC/CAPES, grant n° 14/2020—23072.211119/2020-10; LM, grant n° 88887.469369/2019-00), FINEP (0494/20 01.20.0026.00), and the Rede Corona-ômica BR MCTI/FINEP affiliated to RedeVírus/MCTI (FINEP grant n° 01.20.0029.000462/20 and CNPq grant n° 404096/2020-4), and PPSUS (FAPEMIG, SES/MG, Decit/SCTIE/MS, and CNPq). Fellowships were supported by CAPES (LM, RM-C, and RF), CNPq (CG, RG, MA, TS, and SS), and FINEP (VG, grant n° 1379106).

## Conflict of Interest

The authors declare that the research was conducted in the absence of any commercial or financial relationships that could be construed as a potential conflict of interest.

## Publisher's Note

All claims expressed in this article are solely those of the authors and do not necessarily represent those of their affiliated organizations, or those of the publisher, the editors and the reviewers. Any product that may be evaluated in this article, or claim that may be made by its manufacturer, is not guaranteed or endorsed by the publisher.

## Supplementary Material

Supplementary Figure 1 Form applied before personal sampling collection. The form includes demographic information, medical history (including COVID-19 tests and results), signs, symptoms, travel behavior, and possible contact with SARS-CoV-2 positive person.

Supplementary Figure 2 Workstation surfaces for the environmental testing in dental clinic. Figure shows dental chair area, and the samples site of collection. The black arrow shows a tube kept open during the whole procedure of environmental sampling. Red and yellow lines highlight the areas. The red arrow points to the internal part of the saliva ejector.

Supplementary Figure 3 Purge area and sites collected. Red circles highlight areas where samples were collected.

Supplementary Figure 4 Supporting area and sites collected. Red lines highlight areas where samples were collected. The blue circle shows the position of the tube kept open.

Supplementary Figure 5 Dental care healthcare professionals' RT-PCR results for SARS-CoV-2, according to the presence in the clinic per week. **(A)** Dental students and work pair results. Three students (S#9, S#14, and S#51) had COVID-19 previously and returned to the dental school after this project started on the third and fifth week. All of them reported that they did not contact each other or with their work pair before the start of the study. They started to work at the clinic after negative RT-PCR results. The absence of S#22, and S#28 in the 9th week was because they concluded the undergraduation. **(B)** Teachers' results. L#10 were not present in the 1st week because had contact to a familiar positive to COVID-19. L#4, L#8, L#11, L#15 and L#28 were absent due to personal reasons not related to COVID-19 **(C)** Dental assistants' results. The presence of dental assistants was following company schedule. Both dental assistants positive for SARS-CoV-2 were present in the clinic after dental assistance, when no teachers or students were present. (†), zeta variant identified on samples with Ct <30 to N1 and N2 SARS-CoV-2 genes. (‡) S#55 is the unique student who worked without a partner.

Supplementary Figure 6 IgG and IgM positive results in dental healthcare professionals during the nine weeks observed. **(A)** Shows IgM positive results. **(B)** Shows the number of IgG positive results. **(C)** Demonstrate the double IgM and IgG positive results. Black lines represent cumulative positive results.

Supplementary Figure 7 Environmental results of samples collected from surfaces of each workstation (WS) collected in the eighth and ninth weeks. The figure illustrates different areas detected between the two weeks. The patient positive for COVID-19 was assisted in the eighth week at workstation#2 where indeterminate samples were on the sink bench, detergent dispenser, air sample, instrument table handle, and internal part of the saliva ejector were found (Ct = 35 ± 2 for N1 gene).

Supplementary Figure 8 Phylogenetic tree of variants in Belo Horizonte City and the variants detected in dental healthcare professionals (highlighted in the purple circle). Sequenced samples resulted in a total of 183,560.5 reads with a genome span above 79.5% (mean: 89.6 ± 10.1%). The sequencing depth of the two samples were at least 740x (Mean 1226.0 ± 486).
