# Supplementary figures and images for: Biosafety in Dental Health Care During the COVID-19 Pandemic: A Longitudinal Study
Source: Front Oral Health. 2022 May 10;3:871107. doi: 10.3389/froh.2022.871107 (PMC9127188; doi:10.3389/froh.2022.871107)

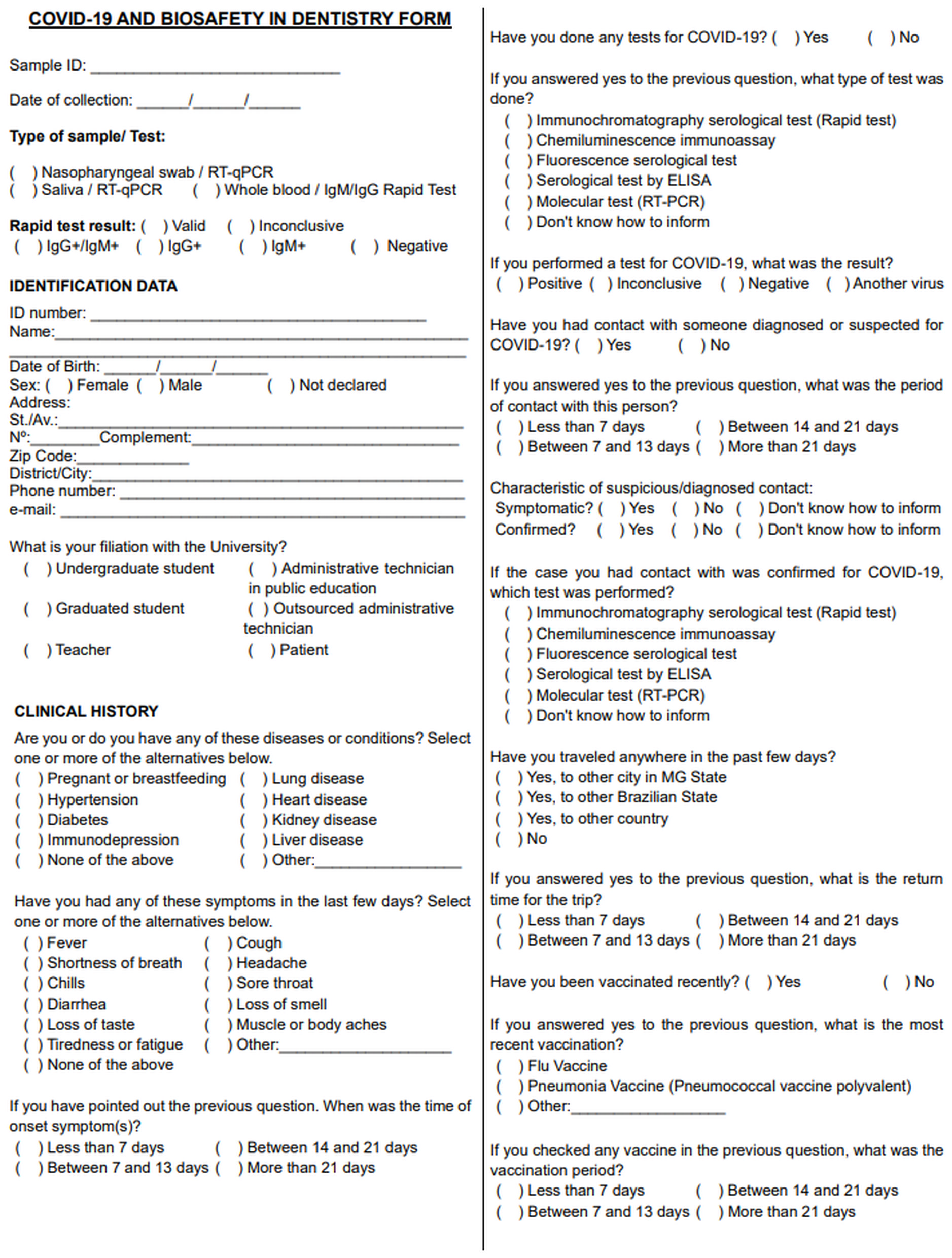

Supplement: Supplementary Figure 1 — Form applied before personal sampling collection. The form includes demographic information, medical history (including COVID-19 tests and results), signs, symptoms, travel behavior, and possible contact with SARS-CoV-2 positive person. [file Image_1.TIF]

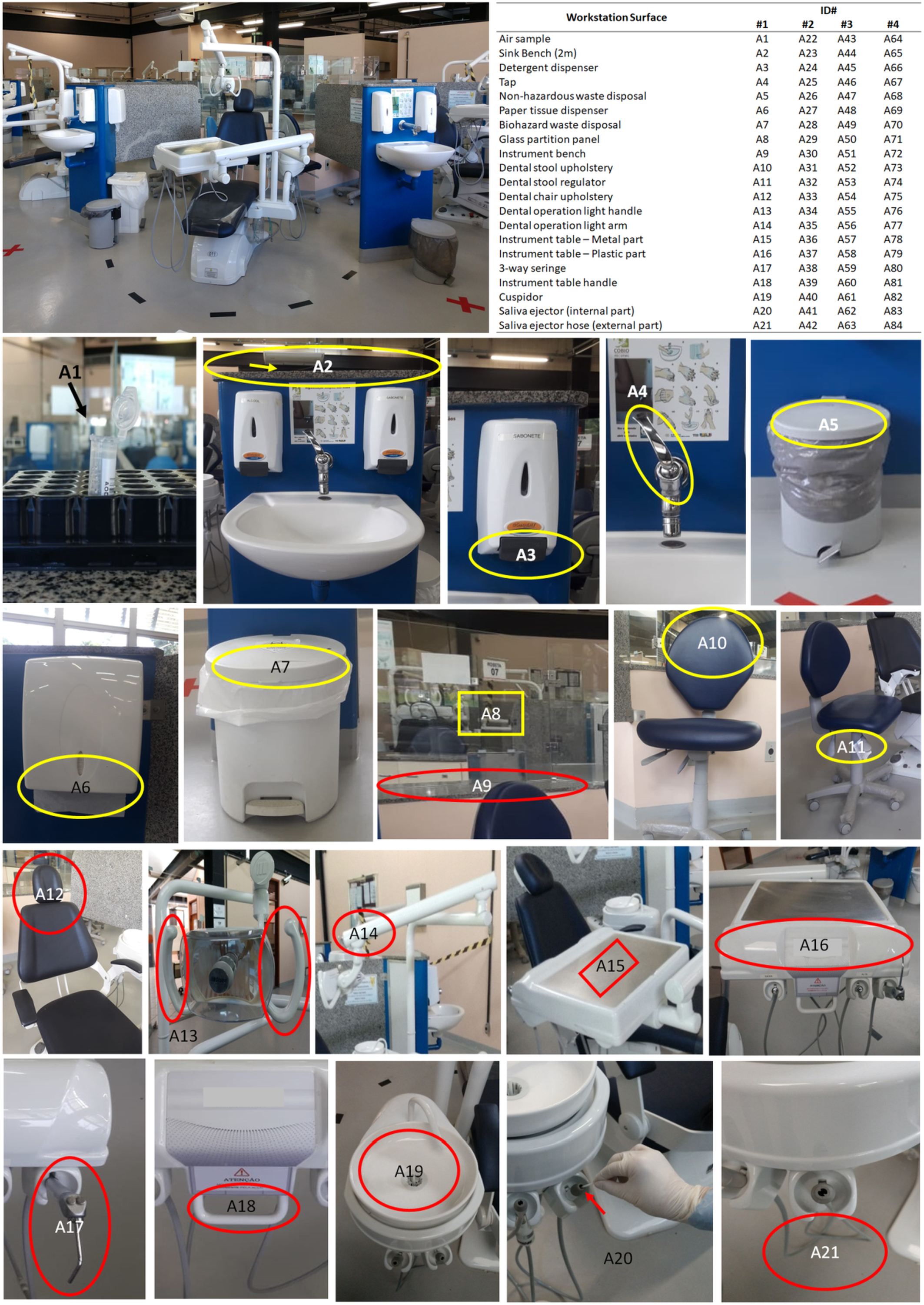

Supplement: Supplementary Figure 2 — Workstation surfaces for the environmental testing in dental clinic. Figure shows dental chair area, and the samples site of collection. The black arrow shows a tube kept open during the whole procedure of environmental sampling. Red and yellow lines highlight the areas. The red arrow points to the internal part of the saliva ejector. [file Image_2.TIF]

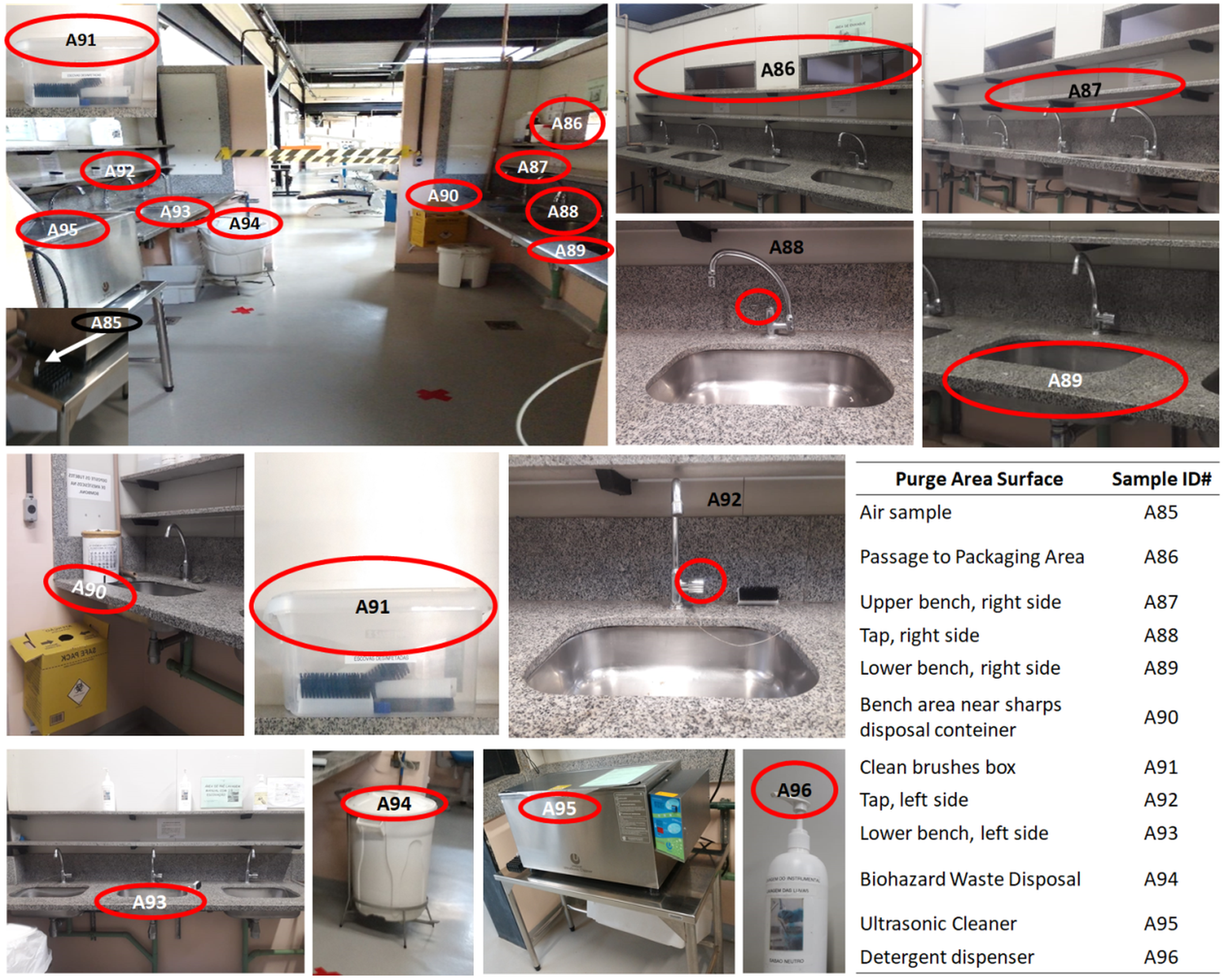

Supplement: Supplementary Figure 3 — Purge area and sites collected. Red circles highlight areas where samples were collected. [file Image_3.TIF]

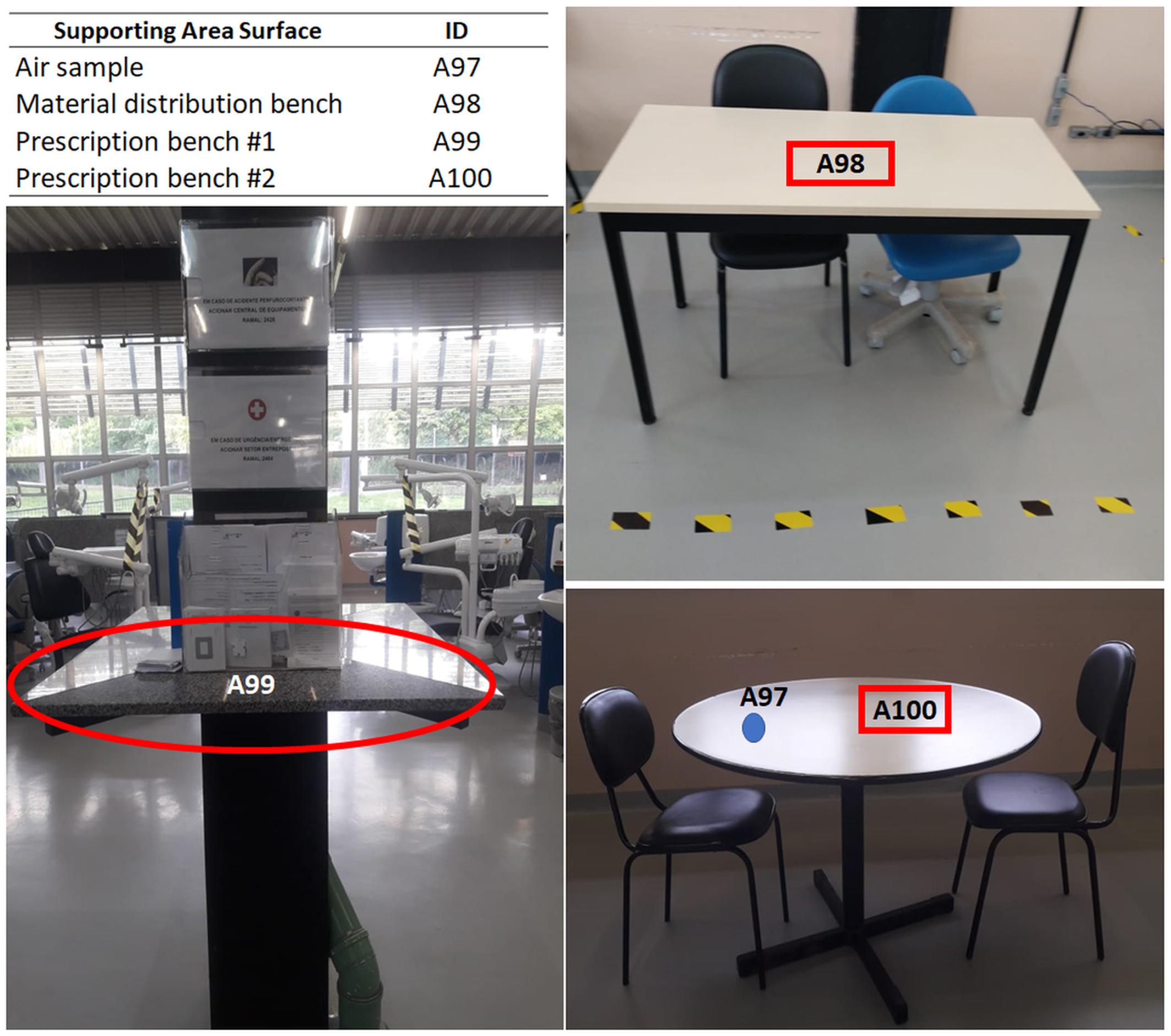

Supplement: Supplementary Figure 4 — Supporting area and sites collected. Red lines highlight areas where samples were collected. The blue circle shows the position of the tube kept open. [file Image_4.TIF]

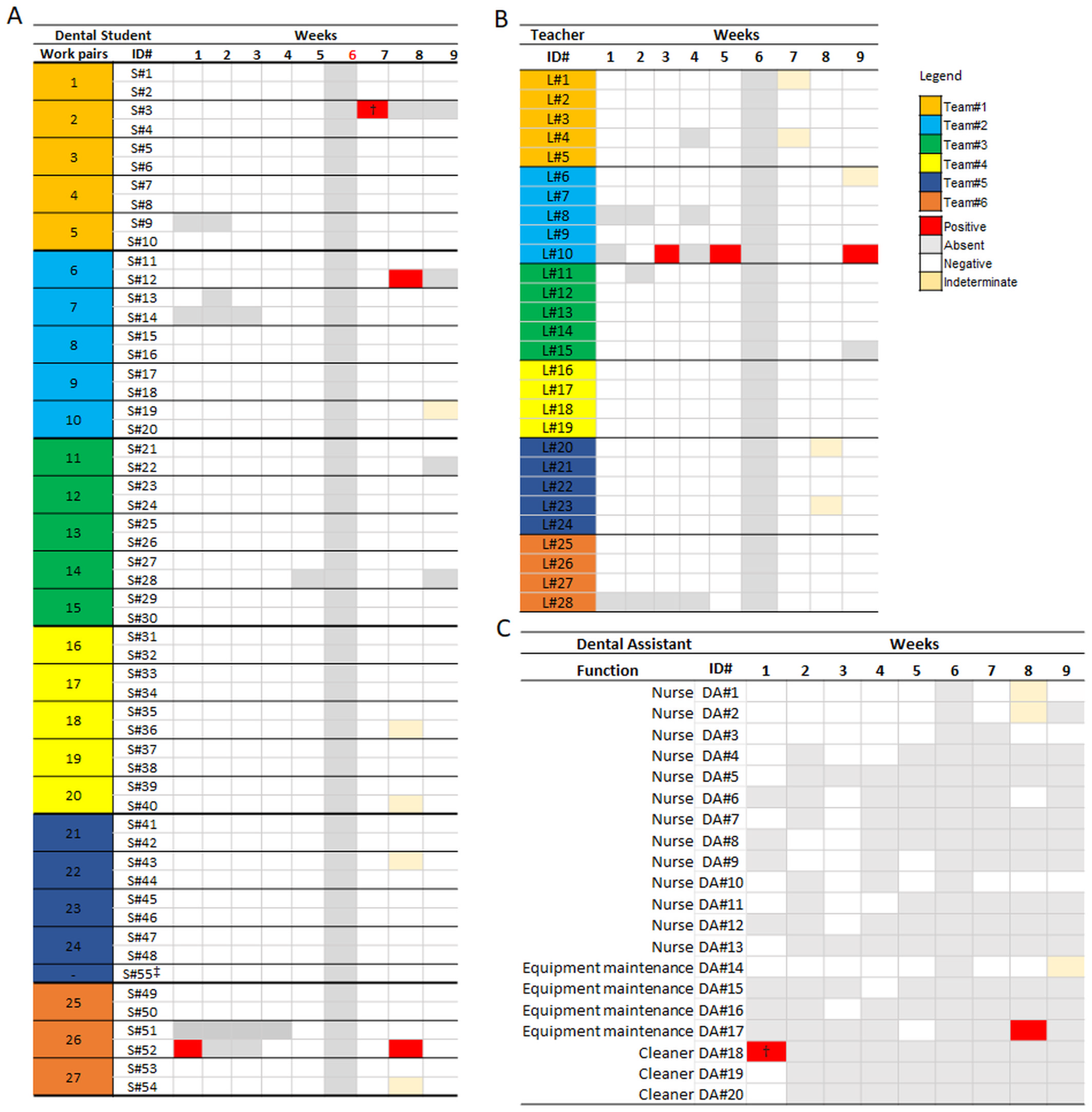

Supplement: Supplementary Figure 5 — Dental care healthcare professionals' RT-PCR results for SARS-CoV-2, according to the presence in the clinic per week. (A) Dental students and work pair results. Three students (S#9, S#14, and S#51) had COVID-19 previously and returned to the dental school after this project started on the third and fifth week. All of them reported that they did not contact each other or with their work pair before the start of the study. They started to work at the clinic after negative RT-PCR results. The absence of S#22, and S#28 in the 9th week was because they concluded the undergraduation. (B) Teachers' results. L#10 were not present in the 1st week because had contact to a familiar positive to COVID-19. L#4, L#8, L#11, L#15 and L#28 were absent due to personal reasons not related to COVID-19 (C) Dental assistants' results. The presence of dental assistants was following company schedule. Both dental assistants positive for SARS-CoV-2 were present in the clinic after dental assistance, when no teachers or students were present. (†), zeta variant identified on samples with Ct <30 to N1 and N2 SARS-CoV-2 genes. (‡) S#55 is the unique student who worked without a partner. [file Image_5.TIF]

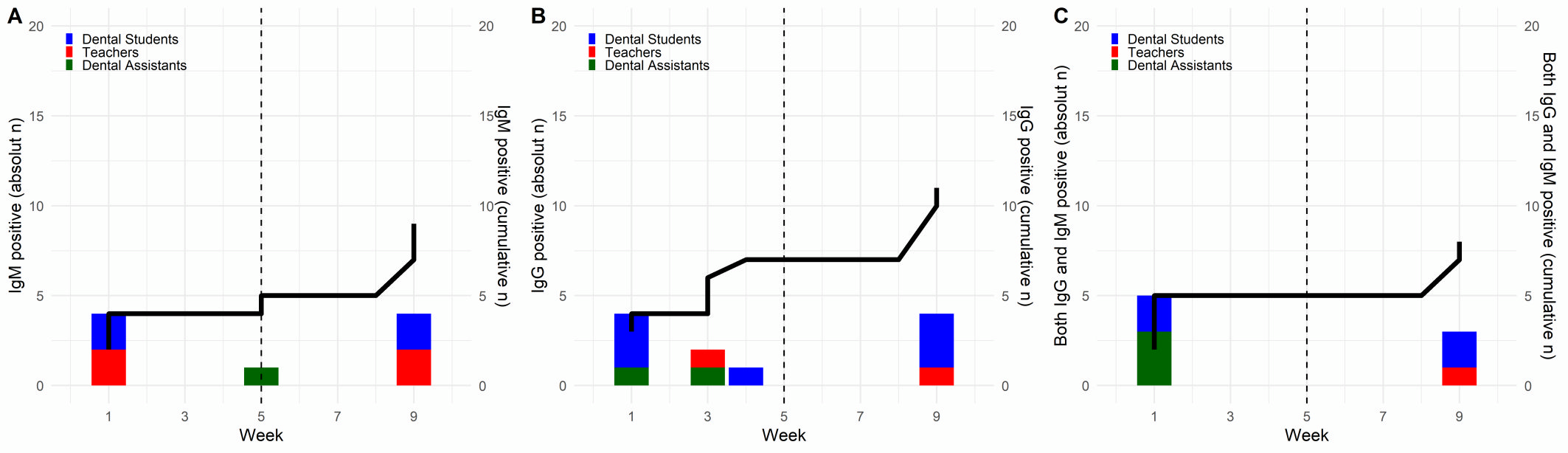

Supplement: Supplementary Figure 6 — IgG and IgM positive results in dental healthcare professionals during the nine weeks observed. (A) Shows IgM positive results. (B) Shows the number of IgG positive results. (C) Demonstrate the double IgM and IgG positive results. Black lines represent cumulative positive results. [file Image_6.TIF]

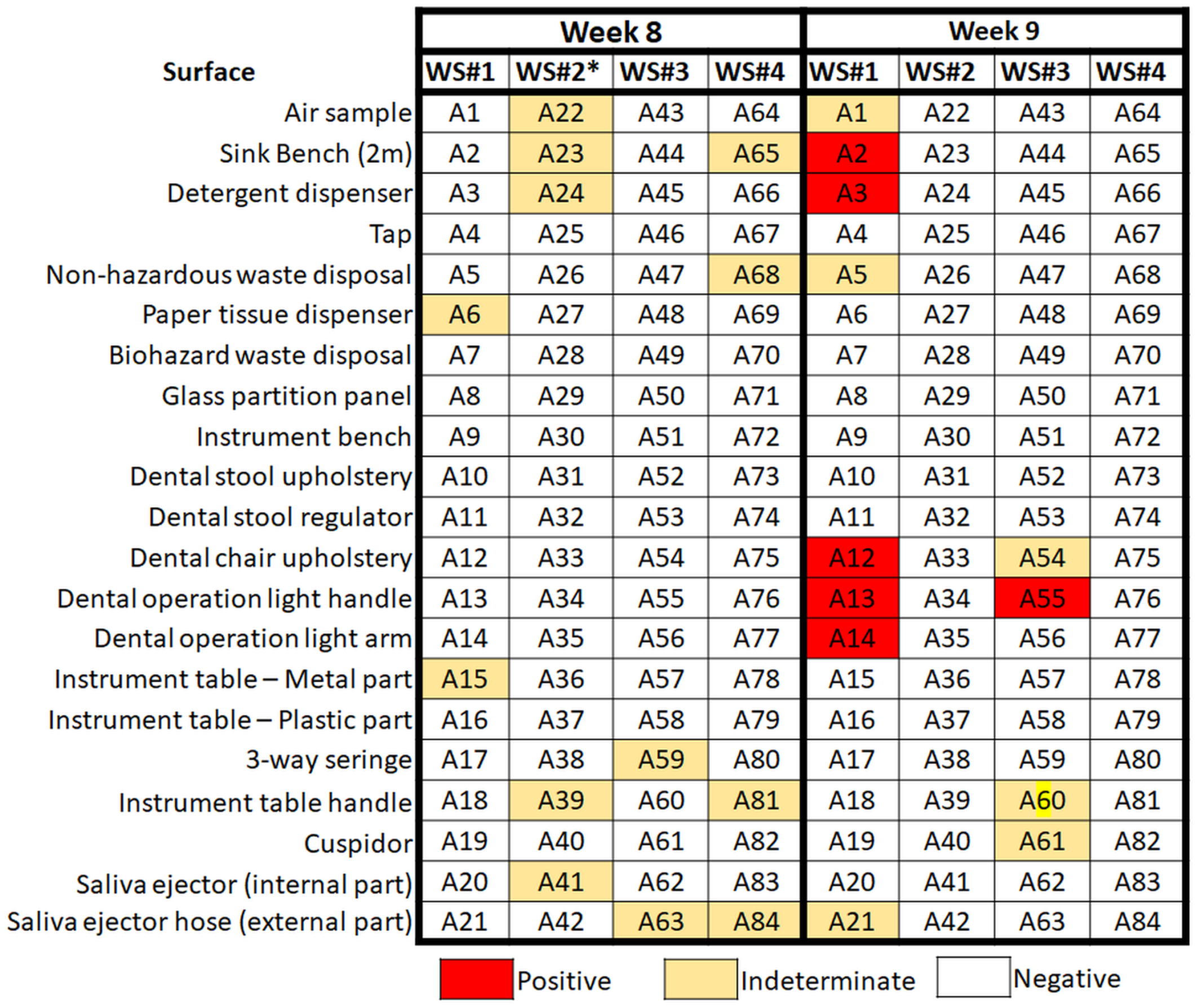

Supplement: Supplementary Figure 7 — Environmental results of samples collected from surfaces of each workstation (WS) collected in the eighth and ninth weeks. The figure illustrates different areas detected between the two weeks. The patient positive for COVID-19 was assisted in the eighth week at workstation#2 where indeterminate samples were on the sink bench, detergent dispenser, air sample, instrument table handle, and internal part of the saliva ejector were found (Ct = 35 ± 2 for N1 gene). [file Image_7.TIF]

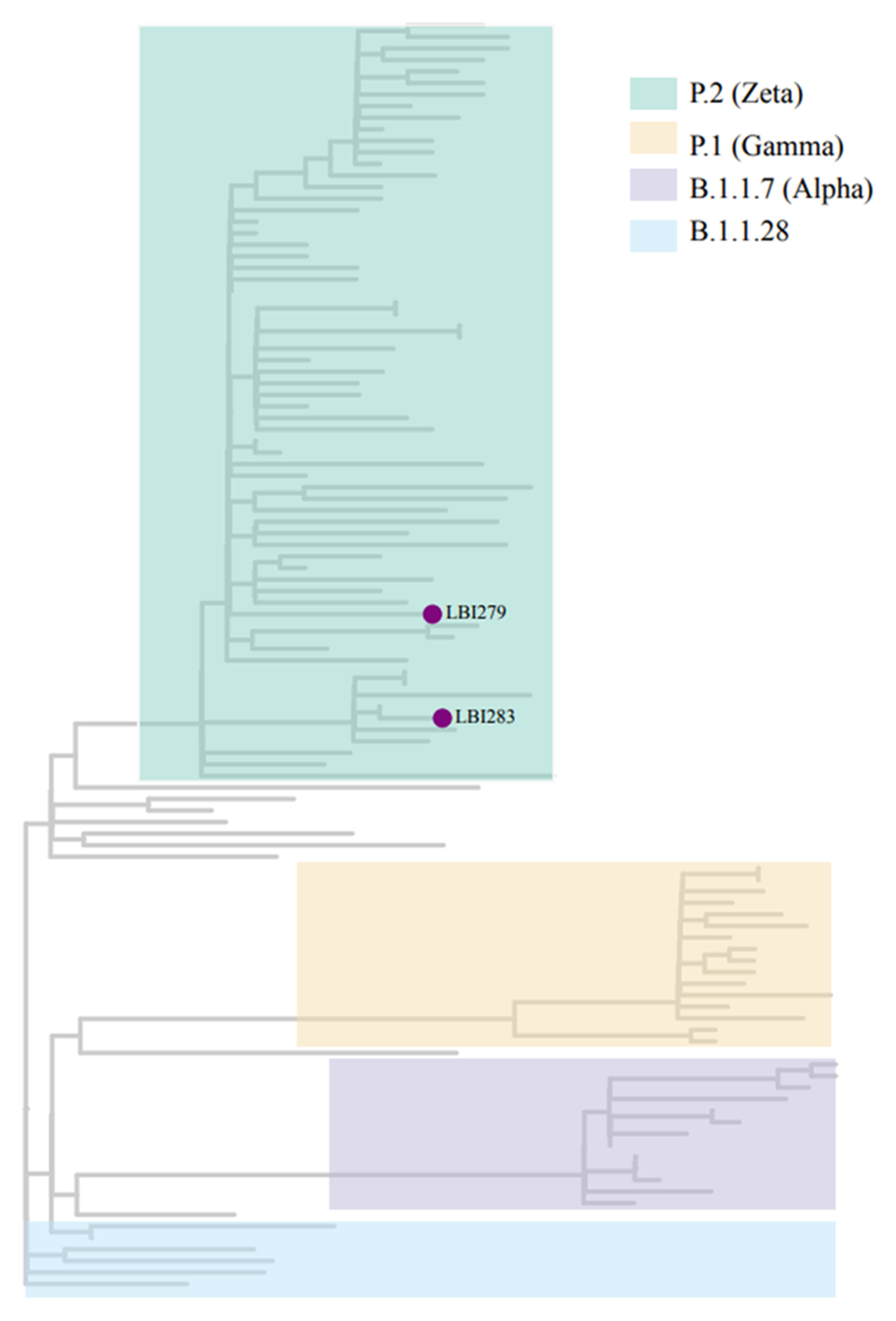

Supplement: Supplementary Figure 8 — Phylogenetic tree of variants in Belo Horizonte City and the variants detected in dental healthcare professionals (highlighted in the purple circle). Sequenced samples resulted in a total of 183,560.5 reads with a genome span above 79.5% (mean: 89.6 ± 10.1%). The sequencing depth of the two samples were at least 740x (Mean 1226.0 ± 486). [file Image_8.TIF]
